# Transcriptomic markers meet the real world: finding diagnostic signatures of corticosteroid treatment in commercial beef samples

**DOI:** 10.1186/1746-6148-8-205

**Published:** 2012-10-30

**Authors:** Sara Pegolo, Guglielmo Gallina, Clara Montesissa, Francesca Capolongo, Serena Ferraresso, Caterina Pellizzari, Lisa Poppi, Massimo Castagnaro, Luca Bargelloni

**Affiliations:** 1 Department of Comparative Biomedicine and Food Science, University of Padua, Viale dell’Università 16, 35020, Legnaro, Padova, Italy

**Keywords:** DNA-microarray, LC-MS, Anabolic treatment, Cattle, Skeletal muscle, Urine

## Abstract

**Background:**

The use of growth-promoters in beef cattle, despite the EU ban, remains a frequent practice. The use of transcriptomic markers has already proposed to identify indirect evidence of anabolic hormone treatment. So far, such approach has been tested in experimentally treated animals. Here, for the first time commercial samples were analyzed.

**Results:**

Quantitative determination of Dexamethasone (DEX) residues in the urine collected at the slaughterhouse was performed by Liquid Chromatography-Mass Spectrometry (LC-MS). DNA-microarray technology was used to obtain transcriptomic profiles of skeletal muscle in commercial samples and negative controls. LC-MS confirmed the presence of low level of DEX residues in the urine of the commercial samples suspect for histological classification. Principal Component Analysis (PCA) on microarray data identified two clusters of samples. One cluster included negative controls and a subset of commercial samples, while a second cluster included part of the specimens collected at the slaughterhouse together with positives for corticosteroid treatment based on thymus histology and LC-MS. Functional analysis of the differentially expressed genes (3961) between the two groups provided further evidence that animals clustering with positive samples might have been treated with corticosteroids. These suspect samples could be reliably classified with a specific classification tool (Prediction Analysis of Microarray) using just two genes.

**Conclusions:**

Despite broad variation observed in gene expression profiles, the present study showed that DNA-microarrays can be used to find transcriptomic signatures of putative anabolic treatments and that gene expression markers could represent a useful screening tool.

## Background

The use of growth promoters in meat production has been banned in the European Union since 1988 due to the potentially adverse effects of hormone residues for the consumer. Council Directive 23/96/EC requires the EU member States to adopt National Monitoring Plans to control the illegal use of these compounds. Despite the ban, these substances are still administered and a black-market for the production, distribution, and use of multiple steroids has flourished [1]. To elude official controls, new anabolic compounds are developed and growth promoters are administered at low doses or combining different substances in hormone cocktails. To keep pace with such a moving target, constant innovation in screening and validation methods is necessary.

Anabolic steroids act on multiple organs and metabolic pathways either through primary interaction or secondary effects. For this reason, indirect approaches, based on the evaluation of perturbations of different biological systems, have been proposed to identify growth-promoter-treated animals. Target organ histology, transcriptomics, proteomics, and metabolomics have been explored as screening tools to better inform confirmative analysis [2-5]. The application of transcriptomics in toxicology has experienced an impressive growth in recent years, leading to the foundation of a new discipline, toxicogenomics [6]. Although toxicogenomics has mostly focused on the effects of pharmacological compounds in model species, it is increasingly applied to monitor the effects of xenobiotics in non-model species (e.g. eco-toxicogenomics). In this contest, gene expression profiles have been used to obtain indirect biomarkers for the use of growth promoters in beef meat production. Initially, quantitative real-time PCR was used to analyze the expression of candidate diagnostic genes. This approach has already been successfully applied in several studies on experimentally treated animals [7-12]. However, good candidate genes are often difficult to identify and the use of single or few gene markers provides a limited and biased view of the biological response to xenobiotics. Using either DNA microarray platforms or RNA-sequencing, it is possible to obtain whole-transcriptome expression profiles, which provide a broad and unbiased (hypothesis-free) picture of the biological response to toxicants. Thanks to the decreasing costs of genomic technologies, transcriptomics is starting to be applied to the identification of gene markers for anabolic treatments in beef cattle. Microarray analysis has been used so far to examine the effects of anabolic hormones in experimentally treated animals, as in the case of skeletal muscle samples from bulls administered with Dexamethasone (DEX) and DEX plus 17β-estradiol [13], and with trenbolone acetate plus estradiol [14], or livers from beef cattle after experimental treatment with dehydroepiandrosterone [15]. More recently, the potentiality of RNA- sequencing technology for the detection of growth-promoters abuse in cattle was explored and successfully used to screen for highly regulated genes to be proposed as biomarker candidates for detecting the treatment with trenbolone acetate plus estradiol [16].

In the present study, the use of transcriptomics is extended for the first time to unknown skeletal muscle samples, collected directly from beef cattle immediately after slaughtering. These samples are part of a larger survey during which also thymus and urine specimens were collected with the aim of detecting putative use of corticosteroids in commercial animals. Histological analyses on thymic samples were used following a validated protocol to classify samples as positive, suspect or negative for corticosteroid treatment and results have been reported previously [17], showing a relevant percentage of suspect animals for corticosteroid treatment compared to official data. Here, detection of DEX urinary excretion by Liquid Chromatography - Mass Spectrometry (LC-MS) was additionally implemented to provide direct evidence of illicit use of anabolic steroids. All samples (LC-MS positive and negative samples, positive, suspect or negative from histological analysis) were analyzed using a cattle-specific oligo-DNA microarray and compared with known negative controls. The goal of this study was to identify gene expression patterns that could classify unknown commercial samples as negative or putative positive (suspect) by comparison with untreated controls.

## Results

### LC-MS analyses

**Figure 1:**
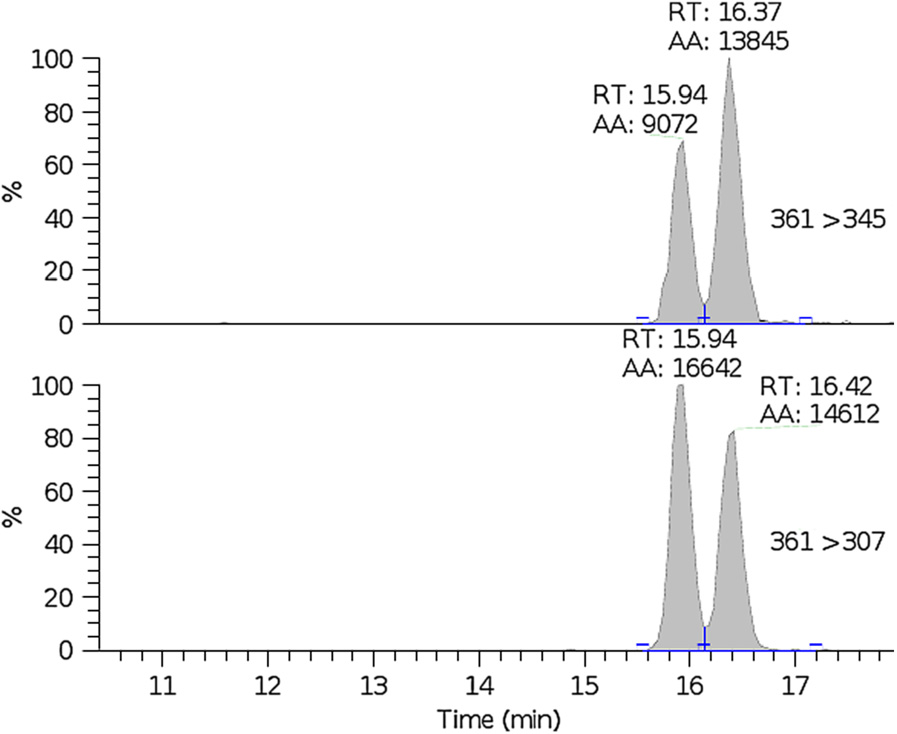
**Specificity of the LC-MS method.** Diagnostic ion chromatograms obtained in bovine blank urine sample spiked with 2.5 ng/mL of betamethazone (retention time 15.9 min) and DEX (retention time 16.4 min).

LC-MS analyses were performed on urine from the slaughtered animals to obtain an analytical confirmation for the thymus histological results [17]. First, the LC-MS method was validated and results of method validation are summarized in Tables 1 and 2. The method validation process for DEX analysis consisted of the analysis of 20 different blank urine samples in order to verify the absence of target analyte and potential interfering compounds. No interferences being detected in the analyte diagnostic chromatograms. Figure 1 reports the extracted ion chromatograms obtained from a blank urine spiked with betamethasone (rt 15.9 min) and DEX (rt 16.4 min) at 2.5 ng/ml, showing the ability of the chromatographic method to separate the peaks of the two isomeric corticosteroids so avoiding the possibility of misrecognition. For all the analytes, the confirmation of the identity according to Decision 2002/657/EC was demonstrated by comparing the relative retention time observed for the spiked analytes to the standards, furthermore two granddaughters ions, with a signal to noise greater than 3, were monitored and all ion ratios of samples were within the recommended tolerance when compared with standards (Table 1).

**Table 1:** Confirmation data of DEX in spiked urine analyzed by LC-MS^3^

**Analyte**	**RR**_**exp**_^**a**^	**RRT**_**req**_	**RRT**_**passed**_	**Ion ratio**_**exp**_^**b**^	**Ion ratio**_**req**_	**Ion ratio**_**passed**_	**LOQ (ng/mL)**
DEX	1.389	1.354-1.423	Y	0.990	0.790-1.190	Y	0.25

^a^ Relative retention time (min).

^b^ Area ratio of product ions from extracted ion chromatograms.

**Table 2:** LC-MS^3 ^validation

**Analyte/parameter**		**DEX**	
spike level (ng/ml)	0.25	0.50	2.50
repeatibility (R.S.D., %)	9.3	9.8	5.6
recovery (%)	89	92	110
linearity (r^2^, 0.20-10 ng/ml)		0.997	
LOD (ng/ml)		0.15	
LOQ (ng/ml)		0.25	
CCα (ng/ml)		0.19	
CCβ (ng/ml)		0.33	

Validation data for the determination of DEX in urine by LC-MS^3^ (n=18 at each level).

**Figure 2:**
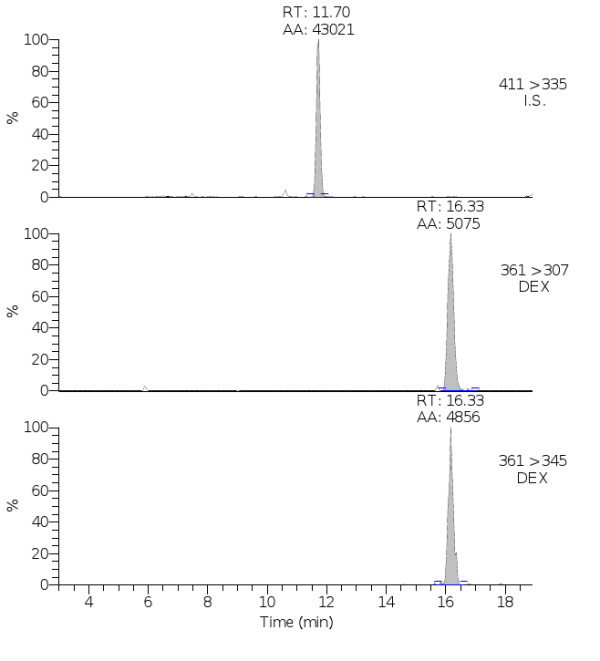
**Diagnostic ion chromatograms obtained from the analysis of the urine sample P21.** IS: internal standard; DEX: Dexamethasone.

The results of LC-MS^3^ analysis confirmed the presence of DEX residues in urine of the four animals positive for histological data (P21, P54, P56, P57; Figure 2, Table 3). The concentration of DEX ranged from LOD and 1.2 ng/ml.

**Table 3:** LC-MS^3 ^analysis for dexamethasone (ng/ml)

**sample**	**DEX (ng/mL)**
**K7**	n.d.
**K8**	n.d.
**K10**	n.d.
**K11**	n.d.
**K12**	n.d.
**C17**	n.d.
**C18**	n.d.
**C20**	n.d.
**C21**	n.d.
**C22**	n.d.
**C23**	n.d.
**C24**	n.d.
**C41**	n.d.
**C44**	n.d.
**C45**	n.d.
**C46**	n.d.
**C47**	n.d.
**C48**	n.d.
**P21**	0.5
**29**	n.d.
**36**	n.d.
**37**	n.d.
**38**	n.d.
**39**	n.d.
**49**	n.d.
**50**	n.d.
**51**	n.d.
**52**	n.d.
**P54**	1.2
**P56**	0.2 ^b^
**P57**	0.4
**58**	n.d.
**60**	n.d.
**61**	n.d.
**76**	n.d.
**78**	n.d.
**79**	n.d.
**81**	n.d.
**263**	n.d.
**265**	n.d.
**266**	n.d.
**269**	n.d.
**270**	n.d.

^a^ value > cut off factor.

^b^ value < LOQ.

n.d.: not detected (< LOD).

Results from urine randomly collected at slaughterhouse.

### Microarray analysis

Microarray experiments for all the 43 bovine skeletal muscle samples were performed.

High quality, unbiased and reproducible gene expression data are always desirable in any DNA microarray experiment, but when the aim is to apply transcriptomics for the identification of illicit use of steroid hormones, data quality becomes essential for obvious reasons. Comparison of normalized, averaged spike-in signals across different experimental replicates provided a strong indication of the robustness of the normalization process as spike-in variation across samples was minimal. The filtering process on the basis of the second lowest spike-in concentration resulted in removing 1,340 unique transcripts. Finally, gene-specific quantitative (q) RT-PCR assays were developed and used to quantify relative expression of 10 genes in the whole set of samples. As shown in Table 4, a positive and significant correlation of expression values was found for all validated genes. The overall correlation of FC values calculated for the two methods (qRT-PCR and DNA-microarray) also showed a high correlation coefficient (Spearman’s rho=0.87; *P *< 0.001).

**Table 4:** Spearman’s rho for the set of selected genes used for qRT-PCR validation

**Gene Symbol**	**Spearman’s rho**	**FC qRT-PCR**	**FC Array**
AMPD1	0.881***	−4.0	−3.0
ANKRD35	0.923***	1.9	2.5
BVLRB	0.598***	1.5	2.8
DDIT4L	0.892***	−3.6	−5.5
GLUL	0.914***	−2.2	−2.3
HOXA9^a^	0.799***	−163.7	−111.9
MEN1	0.504**	1.2	2.0
NAT14	0.846***	2.2	1.6
S100-B	0.902***	1.8	2.0
SIRT3	0.876***	1.8	2.4

^a^ HOXA9 gene expression values evaluated by qRT-PCR in some suspected samples were below the limit of detection and therefore arbitrary values were assigned.

FC was calculated comparing group B *vs* group A animals.

**P < 0.01; ***P < 0.001.

**Figure 3:**
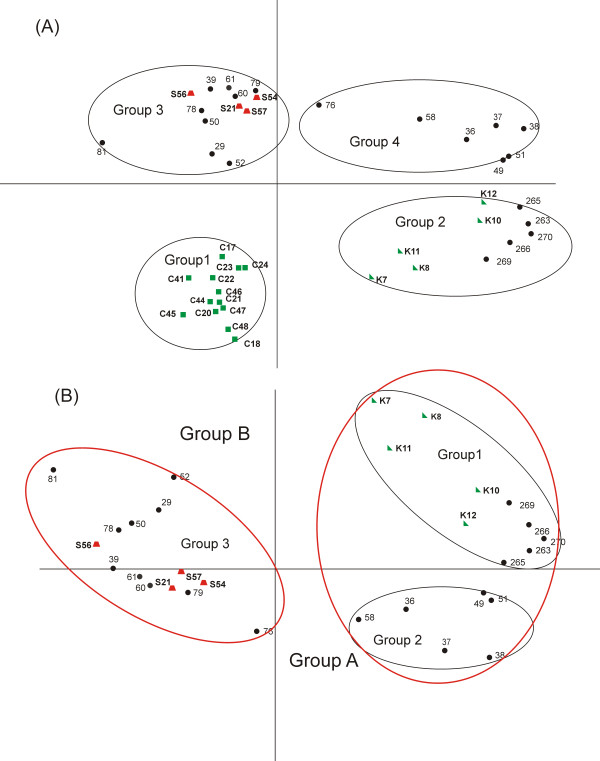
**PCA of the bovine skeletal muscle gene expression profiles.(A)** PCA plot shows the two principal components of greatest variation which cover 42.8% (x-axis) and 28.4% (y-axis) of the total variance. **(B)** PCA plot excluding Holstein samples; x-axis and y-axis cover 56.2% and 14.7% of the total variance, respectively. Green triangles represent one set of controls (K, mixed-breed), green squares the second set of controls (C, Holstein), red trapeziums the animals positive for corticosteroid treatment at histological analyses and LC-MS (P) and black circles the unknown animals.

After data extraction, normalization, and filtering, processed signals for 20,155 unique transcripts in 43 muscle samples were analyzed using PCA. The rationale for using this exploratory statistical tool is based on the need to reduce the inherent multidimensionality of microarray data and to avoid assumptions on the classification of the samples, without prior distinction between negative controls, positive samples, and unknown commercial specimens. The first two components, which account for a substantial fraction (71.2%) of the total variance, clearly identified four main groups (Figure 3A). Negative controls were broadly distributed along the x-axis, which explained 42.8% of the total variance. All the 13 Holstein samples were clustered in group 1, while the remaining five known samples, all cross-breed (Limousine × Charolais), were included in group 2 (Figure 3A). Group 3 included all the four samples (P21, P54, P56, P57) classified as positive for corticosteroid treatment by both histological and LC-MS analyses and the group 4 comprised exclusively samples collected at the slaughterhouse. The two sets of controls were different also with respect to age, as untreated animals in group 1 were animals between 13 and 18 months old, those in group 2 were all 18 months old bulls. To explore the potential association of breed (Holstein, Limousine, Charolais, cross-breed) with the separation of samples along the two principal axes, a one-way ANOVA was carried out using breed as the discriminant variable and individual samples PCA scores either on the x- or the y-axis as dependent variable. A significant partition of samples along both axes (x-axis F = 4.515, p < 0.01; y-axis F = 21.58 p < 0.0001) was evidenced. After excluding Holstein samples, the other breeds were homogenously distributed on both components (x-axis F = 0.225 p = 0.8; y-axis F = 1.585 p = 0.223). Age of animals showed no significant correlation with sample scores on the first component (Spearman rho = 0.21 p = 0.20) and the second one (r = 0.16 p = 0.34). For a few animals the age was putatively approximated during veterinary inspection at the slaughterhouse.

Based on these results, the observed dispersion of negative controls is likely to be attributable to the inclusion of Holstein animals, which is a rather different breed than the French breeds or their crosses that are prevalent in the study. However, breed did not explain the separation of all other samples. Two additional variables that are known to largely affect gene expression in the skeletal muscle, gender and muscle type [18], could be excluded as they were identical for all samples by experimental design.

To avoid possible distortion in the PCA plot due to breed effects, Holstein samples were excluded from the subsequent analyses and the PCA was repeated (Figure 3B). Here, the first two components, which account for 70.9% of the total variance, identified two groups. The x-axis, which explained 56.2% of variance, separated group A, which included the negative controls and some unknown animals, from group B, which comprised all four positive samples and other unknown samples.

To evaluate the statistical significance of the observed clustering, microarray data for the group B were compared to the group A in a two-class unpaired data SAM test. Even enforcing a stringent false discovery rate (FDR, 0%) and a conservative fold-change (FC) threshold (2-fold), a large number of differentially expressed genes (DEG) was obtained, with 2,351 up-regulated and 1,610 down-regulated transcripts (see Additional file 1 and Additional file 2), suggesting a highly significant difference in expression profiles between group A and group B in Figure 3B.

To explore the functional significance of the observed difference, enrichment analysis of DEG was carried out yielding several significantly enriched Gene Ontology (GO) terms and KEGG pathways (Table 5). Different signaling pathways (G-protein coupled receptor protein signaling pathway, cell surface receptor linked signal transduction, olfactory receptor activity) were found to be differentially regulated. In particular, the expression of several genes encoding odorant receptors (ORs) was significantly up-regulated. ORs are expressed in several tissues, but their function outside the olfactory epithelium is largely unknown. However, it has been recently reported that ORs might have a relevant role in myogenesis and muscle regeneration. Griffin and colleagues [19] analyzed 18 OR genes and found that 13 were up-regulated during myogenesis in primary cultured of mouse muscle cells. Most ORs were over-expressed during myoblast fusion. In vivo expression analysis of one OR (MOR23) demonstrated that ORs have an important role during muscle regeneration, guiding cell migration and fusion. In a previous study, Carraro and colleagues [13] examined gene expression profiles of bulls administered with a sub-therapeutic dose of DEX for 43 days in comparison with untreated controls. Within the large set (835) of transcripts that were significantly up-regulated in DEX-treated animals, several genes were found to be involved in regulatory pathways that control myoblast differentiation and skeletal muscle regeneration. Interestingly, the list of up-regulated genes in that study included 13 OR-encoding genes [13], similar to what observed in group B animals in the present work, which showed up-regulation of 56 OR-coding genes.

**Table 5:** GO Biological Process, GO Molecular functions and KEGG pathways analysis of differentially regulated genes

**Category**	**Term**	**Count**	**PValue**	**FE**
GO_BP_FAT	G-protein coupled receptor protein signaling pathway	114	1.44E-05	1.446
GO_BP_FAT	Cell surface receptor linked signal transduction	162	3.14E-05	1.328
GO_BP_FAT	Regulation of transcription, DNA-dependent	125	0.014	1.200
GO_BP_FAT	Regulation of RNA metabolic process	126	0.021	1.182
GO_BP_FAT	tRNA thio-modification	4	0.022	5.332
GO_BP_FAT	Regionalization	19	0.023	1.688
GO_BP_FAT	Pattern specification process	23	0.030	1.552
GO_BP_FAT	Anterior/posterior pattern formation	15	0.031	1.777
GO_BP_FAT	Peptide transport	8	0.037	2.369
GO_BP_FAT	tRNA wobble uridine modification	4	0.048	4.265
GO_BP_FAT	tRNA wobble base modification	4	0.048	4.265
GO_BP_FAT	Pancreas development	4	0.048	4.265
GO_BP_FAT	Positive regulation of organelle organization	8	0.049	2.245
GO_CC_FAT	Intrinsic to membrane	405	2.21E-05	1.157
GO_CC_FAT	Integral to membrane	390	2.86E-05	1.160
GO_CC_FAT	Myofibril	20	8.78E-05	2.511
GO_CC_FAT	Contractile fibre	20	1.84E-04	2.400
GO_CC_FAT	Sarcomere	17	4.27E-04	2.481
GO_CC_FAT	Contractile fibre part	17	8.64E-04	2.354
GO_CC_FAT	Intermediate filament cytoskeleton	18	0.003	2.025
GO_CC_FAT	Intermediate filament	18	0.003	2.025
GO_CC_FAT	I band	11	0.004	2.582
GO_CC_FAT	Keratin filament	10	0.008	2.571
GO_CC_FAT	Z disc	10	0.008	2.571
GO_CC_FAT	Transcription factor complex	22	0.020	1.627
GO_MF_FAT	Olfactory receptor activity	42	9.56E-04	1.629
GO_MF_FAT	Calcium ion binding	95	0.004	1.292
GO_MF_FAT	Passive transmembrane transporter activity	52	0.027	1.312
GO_MF_FAT	Channel activity	52	0.027	1.312
GO_MF_FAT	Substrate specific channel activity	51	0.028	1.312
GO_MF_FAT	Ion channel activity	50	0.033	1.306
GO_MF_FAT	Transcription factor activity	73	0.033	1.236
KEGG	Olfactory transduction	54	2.24E-05	1.725

GO, Gene Ontology; BP: Biological Process; MF, Molecular Function; CC, cellular component; *P* value: modified Fisher exact *P* value calculated by DAVID software; FE, Fold Enrichment defined as the ratio of the two proportions: input genes involved in a biological process and the background information.

DAVID functional annotation of the complete list of differentially regulated genes between group A and group B (see Figure 3B).

Several sarcomere proteins were differentially regulated as well. High doses of DEX were reported to lead to muscle atrophy, mostly affecting fast twitching fibres [20]. A recent study by Stella and colleagues [21], comparing protein expression profiles between beef cattle treated with DEX, alone or in association with clenbuterol, using 2D protein gel electrophoresis and mass spectrometry confirmed that the administration of DEX favors a slow fiber phenotype. Stella and co-workers reported that fast fiber specific proteins (e.g. myosin light chain 1, myosin light regulatory chain 2, and isoform 3 of troponin T) and glycolytic enzymes were significantly under-expressed in DEX-treated animals, while myosin light chain 6B (MYL6B) alkali smooth muscle and non-muscle and isoform 1 of troponin T (TNNT1) were significantly over-expressed. In agreement with such evidence, transcripts encoding TNNT1, MYL6B, and myosin binding protein C slow type were up-regulated in group B animals of the present work while three glycolytic enzymes were significantly under-expressed.

Different myosin isoforms (18B, 7B, 16, 9B), myosin heavy chain 7 (MYH7), 15 (MYH15), 4 (MYH4), myosin regulatory light chain interacting protein (MYLIP) and myosin light chain kinase (MYLK) were up-regulated in group B animals, supporting previous studies showing that glucocorticoids can promote myogenic repair and myoblast proliferation. In particular, while high doses of glucocorticoids in vitro impair C_2_C_12_ myoblast proliferation rate and differentiation capacity, lower doses increase the myogenic fusion efficiency of C_2_C_12_ cells [22].

Also ion channel activity was found to be modified between the two groups of animals. In particular, several genes involved in calcium signaling pathways were up-regulated. It has been reported that Ca^2+^-dependent calcineurin signaling mediates skeletal muscle hypertrophy upon stimulation with IGF1 or insulin associated with DEX [23]. Calcineurin is a calcium-activated protein phosphatase which, upon activation, transduces signal by removing specific phophorylation of the cytoplasmic transcription factor nuclear factor of activated T-cells (NFAT). This allows NFAT translocation to the nucleus, where it activates the transcription of IL-4, a cytokine that has a key role in autocrine/paracrine control of mammalian muscle growth [24]. In fact, IL-4 and IL-4 receptor were found to be over-expressed in group B animals sampled at the slaughterhouse as well as in DEX-treated bulls [13]. Muscle cell membrane depolarization due to K+ efflux has also been reported to activate calcineurin-mediated transcriptional responses [25]. Intriguingly, several potassium voltage-gated channels were over-expressed in group B animals.

The putative similarity in the transcriptomic response in the skeletal muscle of beef cattle after low-dose administration of DEX [13] and in individuals clustering within group B in the present study is statistically relevant. Overall, 206 genes were up-regulated in both sets of samples, showing a highly significant concordance (Fisher Exact test, p < 0.0001). A few “common” transcripts are worth mentioning. Colony stimulating factor 3 receptor (CSF3R) is part of the JAK1-STAT1-STAT3 pathway, which was reported to induce myoblast proliferation [26]. Protein phosphatase 2 (PP2), whose regulatory subunit PP2R1A and catalytic subunit isoform PPP2CA were up-regulated, has an activating role in Wnt signaling, a pathway that was shown to be associated with satellite cell proliferation during muscle regeneration [27]. Actin-related protein 2/3 complex subunit 1A (ARPC1A) is part of a complex that has a critical role in myoblast fusion during either muscle development or regeneration [28]. Elevated expression of Delta-like 1 (DLK1) contributed to hypertrophy in callipyge sheep skeletal muscle [29], while experimental over-expression of DLK1 in mouse skeletal muscle induced hypertrophy [30]. The highly significant concordance with gene expression modifications reported in DEX-treated cattle suggests that all animals of group B might have been illegally administered with DEX. This can explain, at least in part, the observed marked difference in gene expression profiles between group A and group B.

**Figure 4:**
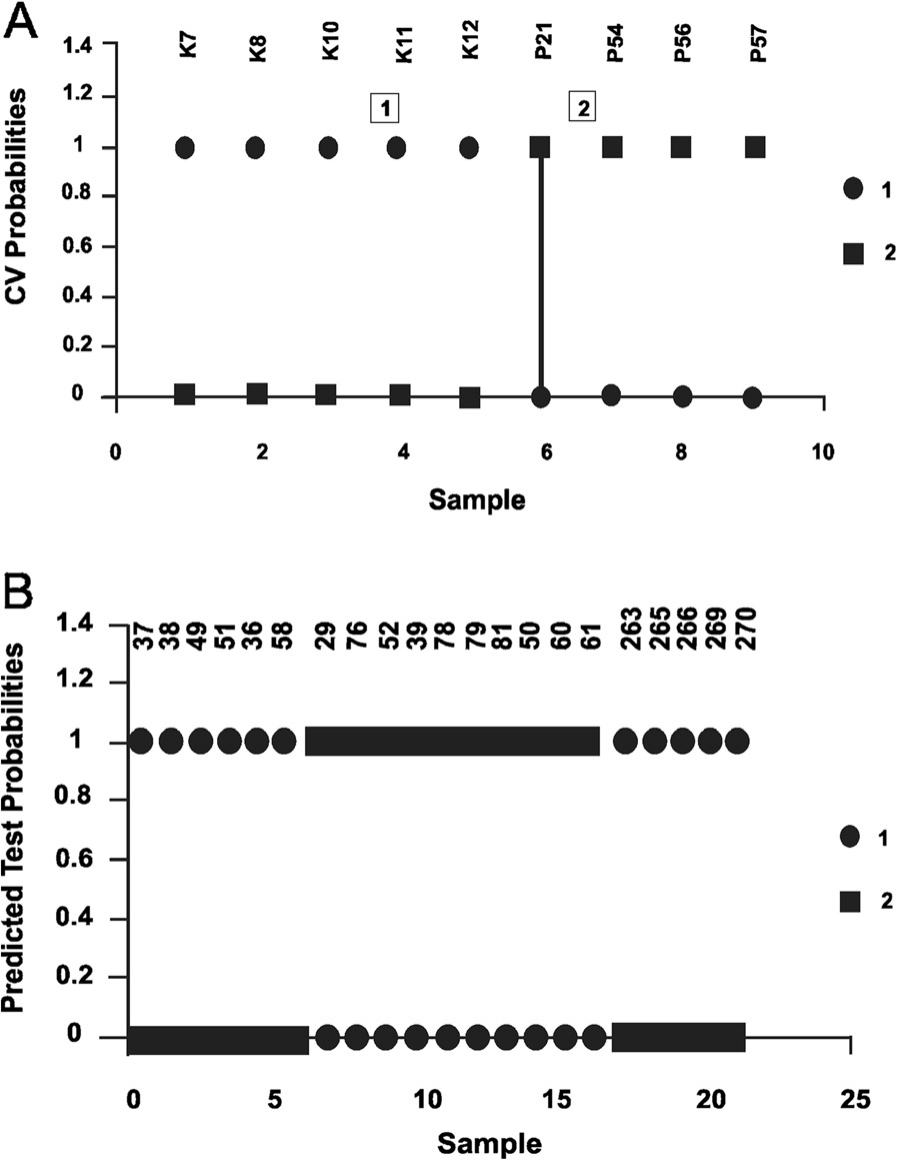
**Plot of cross-validated probabilities and test probabilities for sample classification.(A)** On *x*-axis individual samples: 1–5 negative controls, 6–9 samples positive at histological and LC-MS analyses; on *y*-axis the probability of being classified as controls (circles) or positive (rectangules). **(B)** On *x*-axis individual samples; on *y*-axis the probability of being classified as negative (circles, 1) or positive (rectangules, 2).

Gene expression data from positive samples and negative controls were also analyzed to evaluate the ability to classify unknown samples with a reduced set of informative markers, using a statistical approach for class prediction implemented in the Prediction Analysis of Microarrays (PAM) software. PAM has been extensively applied to classify cancer types based on individual tumor expression profiles. It uses the method of nearest shrunken centroids to find out the minimal set of genes that provides the greatest accuracy of class prediction. The program first performs a discriminant analysis on “known” samples (Training Sample Set) to choose the smallest panel of genes that provide the greatest accuracy of class prediction (the smallest misclassification error). In the present study, the training set consisted of 9 samples, representing two classes, negative and positive. For the former class, the 5 cross-breed control animals were included. For the latter, the 4 samples (P21, P54, P56, P57) that were classified as positive by LC-MS and thymus histological analyses. PAM allowed to exactly discriminate the two classes using only two genes (importin 9, propionyl-CoA carboxylase beta chain, mitochondrial precursor). The accuracy of class prediction using two genes on the Training Sample Set was then estimated through cross-validation (10% of samples were randomly extracted and classified based on the discriminant function calculated on the remaining cases). Figure 4A shows that 100% accuracy was obtained for cross-validation. Finally, the two genes were used to classify all the samples (Test Sample Set) not included in the training set (21 commercial samples). All animals originally clustering in group A were classified as negative, whereas all individuals in group B were predicted as putative positive (Figure 4B).

## Discussion

Two main findings were observed in the present study. First, broad variation in gene expression profiles exists across both known and unknown samples. The causes of such variation are not completely clear, although breed appeared to be the most important. This observation reinforces the obvious, yet often overlooked, need for a most comprehensive representation of biological variation, when indirect biomarkers are applied outside controlled experimental settings. Second, after removing the main source of variation, unsupervised analysis of gene expression profiles showed a highly significant distinction between two groups, one including positive controls and a sub set of commercial samples, the other comprising all negative controls and the remaining unknown individuals. The observed separation was confirmed by a two-class SAM test that identified over 3,900 differentially expressed genes and a class prediction approach that was able to discriminate between the two groups using just two genes, using as a training set positive and negative controls and as test set all unknown samples. Functional annotation of up- and down-regulated transcripts showed several biological processes and molecular pathways that have been already reported in previous proteomic and transcriptomic studies to be altered upon controlled administration of low dosage corticosteroids. Such evidence seems to suggest that unknown animals clustering with LC-MS positive samples might have been administered with glucocorticoids as well. It cannot be completely excluded that all unknown samples in group B showed a transcriptomic profile similar to positive controls for other reasons than being treated with anabolic hormones. On the other hand, such explanation appears quite unlikely as both technical issues (e.g. systematic bias in microarray data) and biological variables known to affect gene expression profiles have been carefully controlled. Therefore, under the working hypothesis that group B samples should considered as suspect, how such hypothesis could be reconciled with the more limited evidence of positive animals (LC-MS) or suspects (histology)? With regard to LC-MS results, one possible explanation is related to the fact that DEX or other corticosteroids are often illegally administered as diluted solution spread on feed and differences in drug uptake can be evidenced across treated animals. Such very low dosages and the rapid metabolism and excretion of these substances make determination of residues extremely difficult [31], even by using targeted GC/MS and LC/MS/MS methods [32-39]. The urinary parent compound and its metabolites concentration are very low during all the treatment time and completely absent a short time after the interruption of the treatment [40], while biological effects, including transcriptomic responses, might still be detectable as such effects are caused by anabolic compounds and often persist after the hormone has been eliminated. It is also well-known that corticosteroids are administered as cocktails of different chemical species with similar biological action to reduce the amount of single compounds. This could make LC-MS less sensitive. In particular, when targeted LC-MS analyses are carried out, for instance searching for DEX, sensitivity is decreased when DEX is not the major component of the cocktail or is entirely substituted with other corticosteroids. With regard to the results of histological analyses, it should be noted that while only four samples could be reliable considered as putative positive, others were considered dubious, including some those included in group B. In fact, the efficacy of the histological method has been recently challenged because of the lack of appropriate reference material considering the evolving nature of animal-rearing practices [41]. Furthermore, administration of corticosteroids leads to thymus cortical atrophy and ‘starry sky’ appearance. However, age-associated thymic involution was evidenced in cattle as in other mammals. As the animals included in the present study were mostly all over 12 months old and most around 15–18 months of age, several positive cases might have been considered negative or dubious because natural thymic involution could not be distinguished from the effects of corticosteroid treatment. On the other hand, the biological effects of corticosteroids on gene expression data were proved to persist several days after time of withdrawal [11,12,42] and thus may evidence the anabolic treatments even when the active compound and its metabolites have been excreted and they are no longer detectable, as mentioned above.

If the working hypothesis of all animals in group B being putative positive were correct, the number of suspect cases would be quite impressive. However, the actual importance of growth-promoter abuse in beef cattle is known to be underestimated [43] because highly informative analytical methods are used on just a small number of cases and often such methods are not sensitive enough to detect residues of low dosage cocktails. This prompts for the development and use of more reliable and cost effective screening tools.

## Conclusions

The present work showed that it might be possible to use just a few gene markers for highly reliable sample classification. However, global transcriptomic tools (DNA microarrays, RNA-seq) are becoming increasingly affordable and rapid, and soon a whole-transcriptome analysis will be feasible in routine practice. Such an approach avoids a priori selection of candidate markers, allows the identification of complex transcriptomic signatures through functional annotation and enables the comparison with other -omic and analytical/chemical studies. This will lead toward meta-analysis of data from controlled experiments as well as field studies and in turn, to the identification of indirect biomarkers less influenced by unknown variables.

## Methods

### Animals and sampling

Tissue samples from a specific anterior limb muscle (*Biceps brachii*) were collected, immediately after slaughtering, from 25 beef cattle randomly selected in 10 different batches and stored in 2 mL RNAlater solution (Ambion, Monza, Italy) at −20°C until extraction. Urine samples were collected during slaughtering from the bladder and each sample, without any preservative, was divided in 15 mL aliquots and stored at −20°C until the analysis. Sample collection was part of a monitoring program on anabolic treatment targeted on indirect biomarkers. The sampling was managed by the Regional Veterinary authorities (Veneto, Italy) and carried out by trained veterinarians. The animals were vaccinated against the main respiratory diseases and treated regularly against parasites typical of the species. They were healthy at *ante-mortem* inspection, passed *post-mortem* inspection, and their meat was approved for human consumption. All individuals were male, their age ranged between 12 and 24 months, and weight between 300–500 kg. The majority of the commercial samples were Charolais, Limousine, or mixed breed (Charolais×Limousine).

Two additional sets of samples from previous controlled experiments were included as negative controls: one set included 5 samples from mixed-breed (Charolais×Limousine) 18 months old male bulls, ~450 kg mean body weight, while the second one included 13 samples from male Holstein beef cattle between 13 and 18 months old, 500–670 kg body weight. The control animals came from experiments conducted according to the guidelines of Italian law (DL 116/92) and European legislation (11 and subsequent amendments) for care and use of experimental animals, and the studies were approved by the Italian Ministry of Health ethical committee.

### LC-MS analysis

#### Chemicals

All solvents for LC-MS were HPLC or analytical grade and purchased from Carlo Erba Reagenti (Milan, Italy). The water used was purified using a Milli-Q system (Millipore, Bedford, MA, USA). Sodium acetate trihydrate and the enzyme for phase II metabolite deconjugation (*Helix pomatia* preparation) were purchased from Sigma-Aldrich (St. Louis, MO, USA). Dexamethasone was purchased from Steraloids (Newport, RI, USA). Cortisol-d4 (internal standard) was obtained from CDN Isotopes (Pointe-Claire, Quebec, Canada).

### Standard solution

Stock solutions of reference standard (1 mg/mL) were prepared in methanol; working solution (1 μg/mL and 0.1 μg/mL) were prepared, monthly, by successive tenfold dilution with methanol and stored in the dark at −20°C. Standard curves were prepared daily by dilution of working solution in water–methanol-formic acid (50:50:0.1, v:v:v) to obtain seven concentration levels ranging from 0.1 to 25 ng/mL.

### Sample preparation

After centrifugation, a 5 mL aliquot of urine was spiked with 25 μL of the internal standard solution (1 μg/mL). The pH was adjusted to approximately 5 by adding 15 ml of 1 M pH 5 acetate buffer, then 50 μL of *Helix pomatia* juice was added to the sample and enzymatic hydrolysis was carried out overnight at 40°C. The preparation was repeated on two separated aliquots, including or omitting the *Helix pomatia* preparation addition and incubation.

Subsequently, 16 mL of the sample was applied to Strata C18-U SPE cartridges (500 mg, Phenomenex, Bologna, Italy) previously activated with 5 mL of methanol and 5 mL of water. After washing with 5 mL of water and 10 mL of methanol–water (20:80, v/v), analytes were eluted with 2 ml of methanol. Then, the extract was applied to Strata NH_2_ cartridges (100 mg, Phenomenex, Bologna, Italy), previously activated with 1 mL of methanol. Purified extracts were evaporated and reconstituted in 500 μL of water–methanol-formic acid (50:50:0.1, v:v:v).

### Liquid chromatography

An Accela 600 HPLC pump with CTC automatic injector was used (Thermo Fischer Scientific, San Jose, CA, USA). Reversed-phase liquid chromatography was performed on a 100 × 2.1 mm i.d., 1.9-μm Thermo Hypersil Gold column. The mobile phases consisted of (A) 0.1% formic acid (v/v) in water and (B) 0.1% formic acid in methanol. The mobile phase composition (A:B; v/v) was: 90:10 at 0 min, 10:90 at 21 min, and 90:10 from 22 to 25 min; the flow rate was set at 200 μL/min. The sample trays was maintained at 4°C. A sample volume of 10 μL was injected.

### Mass spectrometry

Mass spectrometric analysis was performed on a LTQ XL ion trap (Thermo Fischer Scientific, San Jose, CA, USA), equipped with a heated electrospray ionization (HESI-II) probe operating in negative ion mode with the following condition: sheath and auxiliary gas (nitrogen) flow 30 and 10 arbitrary units, respectively; ion spray voltage 2 kV; capillary temperature 275°C; capillary voltage −13 V; tube lens −68 V. Helium was used for collision-induced dissociation.

As already reported [44], in ESI- corticosteroids give an abundant and unique adduct with the conjugated base of the organic acid used; two product ions were obtained employing the formate adduct [M+COOH]^-^ as precursor ion for MS^2^: the pseudomolecular ion [M-H]^-^ and the fragment [M-CH_2_O-H]^-^ which was indicated as corresponding to cleavage of the C21 side chain with loss of formaldehyde [45]. As the [M-H]^-^ ion was not specific, the [M-CH_2_O-H]^-^ ion was used as precursor for MS^3^ to have additional diagnostic ions to obtain a full identification of the analytes. To perform MS^2^ and MS^3^, the precursor isolation was set to 2 Da; precursor ions, product ions and collision energies are shown in Table 6.

**Table 6:** Molecular weights and diagnostic ions of the investigated corticosteroids

**compound**	**MW**	**P.I.(m/z) [M+COOH]**^**-**^	**C.E. MS**^**2**^	**P.I. (m/z) MS**^**2**^	**C.E. MS**^**3**^	**Product ion (m/z) MS**^**3**^
Dexamethasone	392	437	15	361	20	292,307 ^b^,325,345 ^a^
Cortisol-d4 (IS)	364	411	12	335	-	-

C.E. collision energy % - P.I. precursor ion.

^a^ quantifier ion. ^b^ identifier ion.

Xcalibur (version 2.1) data acquisition software from Thermo was used.

### Calibration and quantification

Calibration lines were constructed using pooled urine obtained from samples collected during several experimental plans from bovine of different age and breed.

Pooled urine samples, with no residues of DEX, were spiked with 2.5 ng/mL of IS and with DEX to obtain a concentration range of 0.2-10 ng/mL.

Quantification was based on peak area ratios of the analyte to the IS and a least-squares linear regression analysis was performed to calculate calibration curves.

### Method validation

The validation study was carried out at three concentration levels by the analysis of pooled urine samples spiked with 0.25, 0.50 and 2.5 ng/ml of DEX. To each sample 2.5 ng/mL of the I.S. cortisol-d4 was added, and six replicates of each sample were analyzed on three different days to evaluate the recovery (internal standard corrected) and the precision in term of repeatability (within day) and within laboratory reproducibility (different operators and environmental conditions).

For DEX, the limit of detection (LOD) was calculated as the mean plus three times the standard deviation of the signal-to-noise ratio of 20 representative blank urine samples; the limit of quantification (LOQ) was calculated as the mean plus ten times the standard deviation of the signal-to-noise ratio of 20 representative blank urine samples; the decision limit (CCα) and the detection capability (CCβ) were calculated according to the ISO standard 11843 [46].

### Microarray experiments

#### RNA extraction

Total RNA was extracted from 30 mg tissues using the RNeasy Mini kit (Qiagen, Hilden, Germany) according to the manufacturer’s instructions. The concentration of RNA samples will be measured using a UV–vis spectrophotometer NanoDrop ND-1000 (Nanodrop Technologies, Wilmington, DE) and RNA a was estimated running each sample on a RNA-chip in an Agilent 2100 Bioanalyzer (Agilent Technologies, Palo Alto, CA). RNA integrity number (RIN) index was evaluated using the Agilent 2100 Expert software. RIN is a numerical assessment of the integrity of total eukaroyte RNA samples based on the entire electrophoretic trace of the RNA sample rather than the ratio of the ribosomal bands and allows to standardize the interpretation of RNA quality. In the present study a conservative threshold was enforced in order to reduce experimental biases due to poor RNA quality. To ensure optimal data quality, only RNA samples with RIN number ≥7.0 were included in the analysis as suggested by protocols for microarray experiments.

### RNA amplification, labeling and hybridization

Sample labeling and hybridization were performed according to the Agilent One-Color Microarray-Based Gene Expression Analysis protocol. Briefly, for each sample 200 ng of total RNA were linearly amplified and labeled with Cy3-dCTP. A mixture of 10 different viral poly-adenilated RNAs (Agilent Spike-In Mix) was added to each RNA sample before amplification and labeling, to monitor microarray analysis work-flow. Labeled cRNA was purified with Qiagen RNeasy Mini Kit, and sample concentration and specific activity (pmol Cy3/μg cRNA) were measured in a NanoDrop ND-1000 spectrophotometer. A total of 1,650 ng of labeled cRNA was prepared for fragmentation adding 11 μL 10× Blocking Agent and 2.2 μL of 25× Fragmentation Buffer, heated at 60°C for 30 min, and finally diluted by addition with 55 μL 2× GE Hybridization buffer. A volume of 100 μL of hybridization solution was then dispensed in the gasket slide and assembled to the microarray slide (each slide containing four arrays). Bovine-specific oligo-arrays (Agilent Bovine-Four-Plex G2519F) were used. For most of the transcripts represented on this array, two identical probes are synthesized at two distinct positions on the slide, therefore the average value between the intensities of the two replicate probes was used. The slides were incubated for 17 h at 65°C in an Agilent Hybridization Oven, subsequently removed from the hybridization chamber, quickly submerged in GE Wash Buffer 1 to disassembly the slides and then washed in GE Wash Buffer 1 for approximately 1 minute followed by one additional wash in pre-warmed (37°C) GE Wash Buffer 2. Hybridized slides were scanned at 5 μm resolution using an Agilent G2565BA DNA microarray scanner. Default settings were modified to scan the same slide twice at two different sensitivity levels (XDR Hi 100% and XDR Lo 10%). Microarray data have been deposited in NCBI's Gene Expression Omnibus [47], and are accessible through GEO Series accession number GSE26318.

### Normalization of microarray data

The two linked images generated from the scanned slide were analyzed together, data were extracted and background subtracted using the standard procedures contained in the Agilent Feature Extraction (FE) Software version 9.5.1. The Feature Extraction software returns a series of spot quality measures that enable to evaluate goodness and reliability of spot intensity estimates. Additionally, in each sample, spike-in viral poly-adenilated RNAs were added before sample processing to provide an internal quality control. Each spike-in RNA has a different known concentration following a dilution series and there are 32 replicate probes for each spike-in RNA on the array. All samples (negative, positive, and unknown samples) were then normalized together in a single run to avoid potential biases. After normalization, spike intensities are expected to be uniform across the experiments of a given dataset. Based on the comparison of spike-in probe signal between arrays after normalization, cyclic loess approach was chosen. All control features except for Spike-in (Spike-In Viral RNAs) were excluded from subsequent analyses. After normalization, a further quality control step was performed by removing all probes with intensity values lower than the second lowest spike-in concentration because this value was considered too close to the limit of detection. So, probes with intensity values <4 in at least 30 of the 43 samples were removed from the dataset. Filtering and normalization procedures were performed using R statistical software available at http://www.r-project.org/.

### Quantitative (q)RT-PCR

A set of 10 genes (see Additional file 3) was validated by real-time RT-PCR. This validation step provided independent validation of microarray data. For each selected target gene and for the reference gene (RS5), a (q)RT-PCR assay was designed. Gene–specific primers that encompass one intron were defined for each transcript (except for HOXA9 gene) using the program Primer Express version 2. To design intron-spanning primers, putative intron-exon boundaries were deduced from the Genome Browser Database (http://genome.ucsc.edu/cgibin/hgGateway?hgsid=105081852&clade=vertebrate&org=Cow&db=0).

One microgram of the total RNA for each sample was reverse transcribed to cDNA using Superscript II (Invitrogen, Milan, Italy). An aliquot (2.5 μL) of diluted (1:50 or 1:100) cDNA template was amplified in a final volume of 10 μL, containing 5 μL of KAPA SYBR® FAST Universal 2X qPCR Master Mix (Kapa Biosystems, Inc., Woburn, MA, USA), 0.25 μL of each gene specific primer (10 μM). The amplification protocol consisted of an initial step of 2 min at 95°C, followed by 45 cycles of 3 s at 95°C and 40 s at 60°C. All experiments were performed in a LightCycler 480 instrument (Roche Diagnostics, Milan, Italy). To evaluate the efficiency of each assay, standard curves were obtained amplifying two-fold serial dilutions of the same cDNA, which was used as calibrator. For each sample, the Crossing Point (Cp) was used to determine the relative amount of target gene; each measurement was made in duplicate and normalized to the reference gene RS5, which was measured in duplicate as well.

### Statistical analyses

PCA on normalized and filtered gene expression data was carried out using the TMEV suite [48,49]. Sample classification into two classes was based on visual inspection of PCA plots. A two-class non parametric test for unpaired data was implemented in the program SAM [50], to identify differentially expressed genes between classes, enforcing a FDR of 0% and a FC of 2. Sample class prediction was carried out using the program PAM [51], available online at http://www.stat.stanford.edu/~tibs/PAM. All other statistical tests (ANOVA, Spearman rank correlation test, Fisher exact test) were carried out in R using the RCommander GUI [52].

### Functional annotation

Enrichment analysis on differentially up- and down-regulated genes was performed using the Functional Annotation tool available in the DAVID Database (http://david.abcc.ncifcrf.gov/). GO terms and KEGG pathways included in the DAVID knowledgebase were considered. For KEGG terms, the following parameters were used: gene count 4, ease 0.05. For GO Biological Process, Cellular Component and Molecular Function BP_FAT, CC_FAT, MF_FAT respectively, with gene count 4, ease 0.05 were used.

## Competing interests

The authors declare that they have no competing interests.

## Authors’ contributions

SP was involved in carrying out microarray experiments, microarray data analyses, qRT-PCR validation and writing the draft manuscript. GG carried out LC-MS/MS analyses. CM and FC participated in the study design, analyzed LC-MS/MS, and contributed to drafting the paper. MC contributed to study design and data interpretation. SF and CP were involved in microarray data analysis. LP coordinated sample collection and was involved in data analysis. LB conceived the study, supervised microarray experiments and data analysis, and participated in drafting the manuscript. All authors read and approved the final manuscript. DAVID Database, http://david.abcc.ncifcrf.gov/, 15^th^ May 2012. Genome Browser Database, http://genome.ucsc.edu/cgibin/hgGateway?hgsid-=105081852&clade=vertebrate&org=Cow&db=0, 25^th^ November, 2010.

## Supplementary Material

Additional file 1 Table of the up-regulated genes identified by SAM analysis. Click here for file

Additional file 2 Table of the down-regulated genes identified by SAM analysis. Click here for file

Additional file 3 Table of genes and primers used in the Real-time PCR validation assays. Click here for file
