# Supplementary material for: Transcriptomic markers meet the real world: finding diagnostic signatures of corticosteroid treatment in commercial beef samples
Source: BMC Vet Res. 2012 Oct 30;8:205. doi: 10.1186/1746-6148-8-205 (PMC3541986; doi:10.1186/1746-6148-8-205)
Supplement: Additional file 3 — Table of genes and primers used in the Real-time PCR validation assays. [file 1746-6148-8-205-S3.docx]

**Genes and primers used in the Real-time PCR validation assays**

| **GENE SYMBOL** | **GENBANK ACCESSION** | **PRIMER FORWARD** | **PRIMER REVERSE** |
| --- | --- | --- | --- |
| AMPD1 | XM_590320 | ATGTTCATGCTGGACGTCAGAC | TGCTCCTACGGGATTGTATTTGT |
| ANKRD35 | XM_592376 | CCTCAAGTGTGCTCCTCTTGTGT | GGGTGTACGTCCATCATTATCCA |
| BLVRB | NM_174251 | AATAGGGCTCTGGCCTGTCC | AGGTTTATTGCCCCCTTCCTT |
| DDIT4L | NM_001081519 | AGTAAGAACCCGGCCAGCA | AAATCATTTAGCAGGCTCCCC |
| GLUL | NM_001040474 | CAACCGAAAGCCTGCAGAGA | GCTGGTTGCTCACCATGTCC |
| HOXA9 | XM_865658 | TATGAAACCGCCATTGGGC | GCGTTCAGCCTGATGGAAAC |
| MEN1 | XM_587110 | GCAATGTGCGCGAAGCTCT | CACGGCAGTAGTTGTAGTCCTGG |
| NAT14 | NM_001037245 | GCTGAAGGCTGGCGTGAA | AAGGAGGCCAGGACGAA |
| S100-B | NM_001034555 | TCAGGACGCCGAAACCAGA | CGGCCTTCTCTAACTCAGACATG |
| SIRT3 | XM_874069 | CTGGCCTCGTATTCCAGGTG | CCCCCTCCAGTGTCACTTGA |
| RS5 (reference) | XM_589989 | CATCAAGACCATTGCCGAGTG | CGTAGGAATTGGAGGAGCCCT |
